# Editorial: *South African Journal of Physiotherapy* 2019

**DOI:** 10.4102/sajp.v75i1.1372

**Published:** 2019-09-19

**Authors:** Aimée V. Stewart

**Affiliations:** 1 Department of Physiotherapy, University of the Witwatersrand, Johannesburg, South Africa

The World Confederation for Physical Therapy defines the profession of physiotherapists as follows (WCPT 2015):

Physical therapists provide services for a wide range of people to optimise their physical activity, from elite athletes to aging adults seeking to remain active as they age. More than any other profession, they prevent chronic disease by helping people become more active. Exercise is a principal component of physical therapy programmes designed to increase physical activity. (p. x1x)

For physiotherapists to deliver the high-quality and varied interventions implied in the above statement, they require (Jensen, Gwyer & Shepard 2000):

[*A*] dynamic, multidimensional knowledge base that is patient-centred and evolves through therapist reflection; a clinical reasoning process that is embedded in a collaborative, problem-solving venture with the patient; a central focus on movement assessment linked to patient function and consistent virtues seen in caring and commitment to patients. (p. x28x)

The above statement is linked to the fact that the physiotherapy profession can now be defined as done by Carol Richards in her 2004 ‘Enid Graham Memorial Lecture’ for the Canadian Physiotherapy Association as a clinical science and this is as true now in South Africa as it is in the rest of the world. We have ‘MSc and PhD programmes (research)’; we produce ‘peerreviewed publications’ in this and other international journals, ‘and have a professional journal’ (our journal is listed on PubMed and we are in the final stages of the listing process on Scopus); we have an ‘expanding knowledge base’ with increasing high-quality local inputs; we have increasing numbers of ‘researchers of international reputation’; ‘evidence-based practice’ is emphasised in all our training institutions and is more widespread among our clinicians; but our local ‘knowledge translation into clinical practice’ however needs expansion and improvement (Richards 2004). We must ensure that our expanding knowledge base and translation into clinical practice is done in a way that benefits the majority of our population. If this does not happen, then we cannot claim to be a meaningful part of the healthcare team that is required to deliver high-quality, appropriate and cost-effective healthcare service to the population.

There are insufficient numbers of physiotherapists (total number of active registrations: 7937) to manage the healthcare and rehabilitation requirements of the South African population of 58 780 000) (HPCSA 2019); Statistics South Africa 2019). There are also very few specialised rehabilitation centres for those people who are managed in the public healthcare system. So the rehabilitation requirements of the majority of the population are not being met by our profession, or the healthcare system. Therefore, there is a need for innovative ways in which physiotherapy services can be delivered to ensure appropriate rehabilitation for those requiring it. This will help to improve rehabilitation outcomes for the majority and ensure that our profession plays a meaningful role in meeting the rehabilitation requirements of the population. So what can we, as a profession, do to provide appropriate and meaningful interventions that take into consideration the difficulties that patients within the public healthcare system experience?

We are now at a stage where many of our universities have developed their researchers, to the point that we have increasing numbers of internationally recognised researchers at post-doctoral levels who can make an impact with large, innovative and appropriate intervention studies for those who currently have little or no access to rehabilitation. These researchers should work collaboratively with experienced clinicians to develop management strategies that can be adapted to the needs of the many people who are not able to access rehabilitation. We need to consider adapting that which we do so well at an individual patient level, in a way that provides good-quality rehabilitation care that is affordable, accessible and realistic for those patients who do not have the means to access one-on-one care. We need to test and develop new strategies in a collaborative and multi-centred way to provide appropriate rehabilitation for the public healthcare system.

Already there are some useful studies in the international literature, and some published South African studies with innovative rehabilitation ideas, that could be tailored for use in our communities. These studies now need to be considered for translation into practice and this is where the researchers and clinicians really need to work together to introduce these new ways of practicing to the physiotherapy community working in the public healthcare sector.

The South African physiotherapy profession has the ability to become a leader in these types of innovative studies. Given the dire need of appropriate rehabilitation for the majority of people with chronic diseases and long-term disability in low- and middle-income countries worldwide, let us become involved in innovative rehabilitation studies.

‘I can do things you cannot, you can do things I cannot; together we can do great things.’

– Mother Theresa
